# The Chaeli Campaign Journal Club: Strengthening evidence-based practice and contributing to practice-based evidence in under-resourced South African communities

**DOI:** 10.4102/ajod.v11i0.943

**Published:** 2022-05-18

**Authors:** Rosemary Luger, Martha Geiger, Olwethu Nqevu, Ann Bullen, Faizah Toefy

**Affiliations:** 1 Centre for Disability and Rehabilitation Studies, Faculty of Medicine and Health Sciences, Stellenbosch University, Cape Town, South Africa; 2 The Chaeli Campaign, Cape Town, South Africa

**Keywords:** journal club, interdisciplinary, evidence-based practice, practice-based evidence first person action research, community rehabilitation, under-resourced communities

## Abstract

**Background:**

The Chaeli Campaign is a Cape Town based non-profit organisation offering programmes largely for children and youth with disabilities in diverse under-resourced communities in South Africa. Their therapy team established a Health Professions Council of South Africa accredited interdisciplinary journal club in January 2012, with the aim to improve the team’s service to the community.

**Objectives:**

Our first objective was to make our practice more evidence-based through reading systematically and critically in our field. Our second objective was to write up and share some of our practices to contribute to the generation of practice-based evidence.

**Method:**

First-person action research was applied. The core group of participants over time comprised two occupational therapists, one physiotherapist, two speech therapists, two teachers and four community development workers. Nine iterative cycles of planning, action, review and revised planning have been implemented on an annual basis in this non-formal, long-term action research project.

**Results:**

For over nine and a half years we have pre-read, discussed and completed evaluation questionnaires on 54 peer-reviewed journal articles, conducted 12 conference presentations and published three articles in accredited journals. Participants reported a broadened understanding of issues around disability, more reflective, contextually and culturally appropriate practice and improved interdisciplinary teamwork.

**Conclusion:**

The Chaeli Campaign journal club has built the capacity of therapists, teachers and community development workers to find, read, evaluate and use research evidence to improve their practice. It has also given participants the opportunity to ethically research, present and write up their grass roots interventions, thus contributing to locally applicable practise-based evidence. It is hoped that the sharing of our experience will assist and encourage other teams to start interdisciplinary journal clubs as a step towards facilitating two-way knowledge translation from evidence to practice and from practice to evidence.

## Background

Evidence-based practice is a fundamental underpinning of professional ethics in the rehabilitation field (Buchanan 2011; Chabon, Morris & Lemoncello 2011; Olsen et al. 2013). Therapists registered with the Health Professions Council of South Africa (HPCSA) are required to accumulate continuing professional development (CPD) points to ensure that their clinical skills and knowledge is up to date (HPCSA 2017). The Chaeli Campaign therapists and teachers established an HPCSA accredited interdisciplinary journal club in January 2012 to improve the service offered to communities.

The Chaeli Campaign is a Cape Town based, non-profit organisation (NPO) striving to optimise the inclusion and participation of children and youth with disabilities in context and age-appropriate activities. The therapy team, which in 2022 includes four community development workers (CDWs), an occupational therapist, a physiotherapist, a speech therapist and two teachers work collaboratively and meet formally on a quarterly basis. We have worked in a variety of under-resourced communities, largely in the Western Cape province of South Africa with a focus on the interdisciplinary rehabilitation of children with severe disabilities, carer training, situation-specific inclusive education support where we work alongside children, their families, schools and communities and preventative early childhood development (ECD) stimulation programmes with ECD teachers, classes of children and their parents.

Across the health and rehabilitation sciences, the gap between clinical practice and theoretical developments has been a concern for some time, as evidenced by both earlier and more recent sources (Duncanson, Webster & Schmidt 2018; Frantz & Smith 2013; Zipoli & Kennedy 2005). Journal clubs have been identified as a successful means to enhance evidence-based practice and thus close this gap (Davis et al. 2014; Phillips & Glasziou 2004). There is also a need for more practice-based evidence, that is, research and publications about interventions, programmes and case studies in the actual contexts that practitioners work (Department of Health 2011; Murray & Newton 2008; Straus & Haynes 2009).

The main aim of starting a journal club was to improve the team’s services to children and youth with disabilities.

## Objectives

Our first objective was to make our practice more evidence-based through systematically and critically reading and discussing practical applications of published peer-reviewed journal articles in our fields.

**FIGURE 1:**
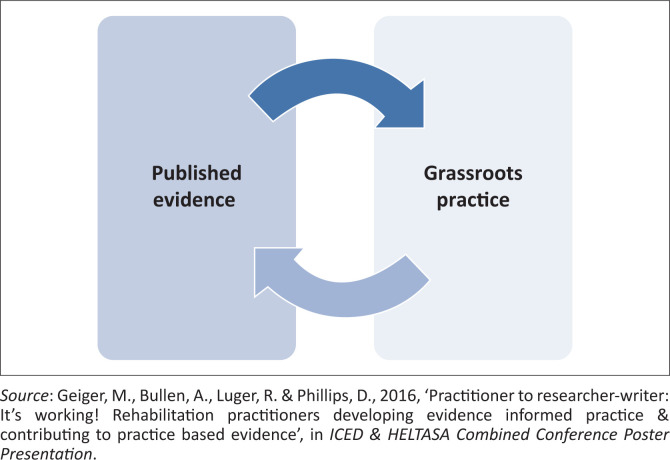
Diagrammatic presentation of our two interdependent objectives.

Our second objective was to contribute contextually generated and locally applicable evidence by sharing and writing up some of our grassroots practices (Figure 1).

## Method

First-person action research (Marshall 2016) was applied as we, the practitioner-participants became the researchers. The choice of the first-person action learning approach was determined as participants became the reflexive researchers in the iterative cycles of planning, implementing and revised planning (Marshall 2016; Zuber-Skerritt 2015). Whilst there have been a few changes in participating staff members over the years and students and guests are included at times, the core group of participants over almost 10 years have comprised two occupational therapists, one physiotherapist, two speech therapists, two teachers and four CDWs, all of whom are female and whose ages range from 25 to 60 years. Nine iterative cycles of reflective and participatory planning, action, review and revised planning have been implemented and are reflected on during the final meeting of each year to ensure that our journal club remains responsive to the changing needs of the therapy team.

### Ethical considerations

As a first-person action research project, the researchers were the participants themselves and formal consent was waived. The authors had obtained formal prior permission from their employer to conduct this journal club and the therapy team was invited by the therapy coordinator and expected to participate in the journal club as part of staff development. The CPD accreditation of the journal club meetings (which carries no cost to the participants) and the learning value of the discussions appear to motivate regular attendance with very few exceptions, only in cases of emergency. It is encouraged, but optional, whether staff facilitates sessions or involves themselves in article writing and presentations.

### Praxis

The initial cycle in 2012 was facilitated by the speech therapist-academic and therapy team coordinator and included registering as an HPCSA interdisciplinary journal club. Since 2013 therapists have taken turns to facilitate discussion meetings and compile the evaluation questionnaire, which consists of 10 questions (multiple choice, true–false and open ended). To upskill themselves the authors participated in in-house tutorials and/or mentoring sessions on sourcing and evaluating articles, basic research methods, principles of ethics and questionnaire design (as required for the evaluation of reading in the journal club meetings for CPD points for the HPCSA-registered therapists). The Chaeli Campaign’s CDWs joined the journal club in 2015. To accommodate varying levels of academic literacy amongst our team, the facilitating therapist provides a summary of the main points at the start of sessions and those not needing CPD points can choose to complete the evaluation questionnaires in pairs to reduce any unnecessary stress. Community development workers have played an active role in preparing for and co-facilitating journal club meetings with therapists since 2018.

Prior to the COVID-19 pandemic the authors met face-to-face (indoors) six times per year. More recently we have continued to meet six times per year, either remotely through Zoom and WhatsApp or in-person outdoors with masks on and physical distancing, depending on the fluctuating lockdown restrictions.

All team members are supported to choose relevant articles. At the end-of-year meeting the authors explore topics and articles they would like to discuss at future meetings. The authors choose six articles for the following year, which meet the HPCSA accreditation criteria (e.g. not older than 5 years) and which are preferably South African or related to our grassroots work in a relevant way. Thus, regular topics include inclusive education, ethical issues, community participation of children with disabilities, the impact of therapy programmes, the lived experience of families with children with various disabilities (intellectual disability, foetal alcohol syndrome, autism spectrum disorder, communication impairment, spina bifida, cerebral palsy), cultural issues, South African health systems, disability studies, sexuality, the impact of poverty and youth development. At the end of each journal club session, the three open-ended questions in the evaluation questionnaire compiled by the presenting therapist encourage application of learning into their current work to ensure that they have contextualised the articles and can leave with the new knowledge translated into practical skills and competencies to use in their work.

It was a huge learning curve for the therapists and teacher involved in writing and getting articles recording some of our collaborative learnings from grassroots work in various under-resourced communities published, as only one member of the team had academic work experience at the beginning. The authors began with areas of work familiar to the whole team and often they first shared their work at a conference as a poster or oral presentation. Then, the article writing task was divided into individual small steps, possible target journals were explored and ethical requirements were carefully considered before writing, submitting and refining the articles based on reviewer comments. The authors persisted with this process as they had struggled to find simple, practical peer-reviewed articles in open access journals, which is essential considering that the vast majority of people who work alongside persons with disabilities do not have high academic and research literacy levels.

Our most recent reflective review took place in the form of a request by the therapy team coordinator in April 2020 to the current therapy team comprising three therapists, two teachers and four CDWs to send written replies to three questions, that is:

1. What are some of your highs and lows of taking part in the journal club?
2. How has this journal club influenced your work?
3. Has the experience of being part of the journal club led to anything further for you?

Three therapists (Therapist 1, 2 and 3), one teacher (Teacher 1) and two CDWs (CDW 1 and 2) chose to respond and excerpts from their verbatim quotes are included in the findings and discussion section following a process of thematic analysis by the interdisciplinary team of authors.

## Limitations of the study

This was primarily a long-term learning and collaborative capacity development activity and expectations of rigorous research could not be applied. However, recommendations for future research can be made.

## Findings and discussion

For over nine and a half years, participants have pre-read, discussed and completed evaluation questionnaires on 54 peer-reviewed journal articles related to our work.

The authors have made 12 conference presentations and published 3 articles in accredited journals. ‘A journey towards inclusive education: A case study from a “township” in South Africa’ (Luger et al. 2012) focuses on two young boys with physical impairment. The authors worked alongside their families and local schools. This was considered important to publish as the creation of facilitating environments and development of open-minded communities is often neglected and results in failed attempts at inclusive education. ‘Parents as partners: Building collaborations to support the development of school-readiness skills in under-resourced communities’ (Pitt et al. 2013) shares a programme that was developed and is still running. It evolved from a need expressed by teachers to have better working relationships with parents and our acknowledgement of how crucial parents are in addressing barriers to learning early on. ‘Simple ideas that work: Celebrating development in persons with profound and multiple disabilities’ (Bullen et al. 2018) outlines some practical suggestions, which were well-received by this usually under-represented population and their caregivers. The simple ideas can be mixed and matched in home and residential settings with children or adults and are all doable in low-resourced environments.

Participants noted some challenges related to participating in journal club, which are important to be aware of and to mitigate against:

‘An initial low was how daunting it was to read more than the abstract and a more recent low is around not always being able to make journal club a place that everyone feels comfortable and wants to be a part of.’ (Therapist 1, female, 40 years old)

‘When I started at the Chaeli Campaign, journal club was the most scary thing for me. The name I think is quite intimidating and the thought of drawing up the questions was very scary for me. Then the first journal club that I had to facilitate was quite daunting.’ (Therapist 2, female, 51 years old)

However, encouragingly, participants report a broadened understanding of issues around disability:

‘Somewhere somehow the topic that we discuss does match with some of the challenges that I come across with my clients. They help me to respond better to the challenges and they also expand my knowledge and understanding on disability matters.’ (Community development worker [CDW] 2, female, 35 years old)

‘My work has been influenced as we have covered numerous relevant topics, which have expanded my skill set and I feel confident to look for and read journal articles that relate to a topic of interest after much graded guidance over many years from [*speech therapist-academic*].’ (Therapist 1, female, 40 years old)

Another benefit reported by participants was of more reflective and contextually relevant practice:

‘It has influenced my work in the way that I am more aware of best practice elsewhere and that there is usually a whole lot more than just therapy per se. Whether it is community entry, working with specific communities, development, context in different situations. I often remember something that we spoke about in a journal club when I am planning something new or revisiting what I am already doing.’ (Therapist 3, female, 56 years)

‘It really has made me think deeply about things that I do automatically which is not necessarily relevant anymore. It is not an easy process to shift one’s thinking, habits and way of doing things, but journal club has given me the opportunity to do this. Journal club has also made me shift my way ofapproaching therapy. I was trained many moons ago from a very top-down Western medicine approach. Many of our journal articles have challenged this old traditional way. So, I have been reminded that my approach to therapy must be more centred around the needs of the child and family andfor it to be culturally relevant.’ (Therapist 2, female, 51 years old)

Improved interdisciplinary team work was also highlighted by participants:

‘The people in the journal club selflessly share skills I don’t have and directly and indirectly, have moulded me into a better community worker. During journal club meetings over the years, we’ve shared challenges and the therapists, teachers and other community workers always help by figuring out ways, in a discussion that includes me, how my work challenges can be resolved. During these meetings I’ve also been exposed to other people’s field experiences, which even if I don’t particularly need to learn them at the time of discussing, I can always go back to and retrieve if/when the challenges arise.’ (CDW 1, female, 34 years old)

‘I think my high is how the journal club in many ways has cemented our relationships with each other. We learn about how other members of the team approach their work in the context of what is being discussed even if we are not directly involved in the same project.’ (Therapist 3, female, 56 years old)

‘It always surprises me how snippets from every article can be related to work at [*school*] even if at first it seems very academic and not really to do with ECD. It’s when we start discussing it that you see how it can be put into practice.’ (Teacher 1, female, 60 years old)

## Conclusion

The Chaeli Campaign journal club has built the capacity of therapists, teachers and CDWs to find, read, evaluate and use research evidence to improve their practice and to ethically research, present and write up grass roots interventions. Participants report a broadened understanding of issues around disability, more reflective, contextually and culturally appropriate practice and improved interdisciplinary teamwork.

It is hoped that the sharing of our experience will assist and encourage other teams, whether at NPOs, community healthcare centres or rehabilitation centres in under-resourced communities, to start interdisciplinary journal clubs as a step towards facilitating two-way knowledge translation from evidence to practice and from practice to evidence with the ultimate aim being to improve the services offered to communities. Furthermore, more formal and rigorous research into action learning methodology and the impact of such a journal club and writing project is recommended.
